# Acromial Osteotomy for Dynamic Posterior Shoulder Instability

**DOI:** 10.1002/atn2.70026

**Published:** 2026-04-30

**Authors:** Ricardo Mendes, José Carlos Garcia Jr, Matheus Barcelos, Airthon Correia, Lucas Fiori, Mauricio Raffaelli, Marcelo Mello

**Affiliations:** ^1^ NAEON Shoulder and Elbow Institute and Sports Medicine Institute São Paulo Brazil; ^2^ NAEON Institute São Paulo Brazil

## Abstract

Posterior shoulder instability is less prevalent than anterior instability but shows a rising incidence, particularly among young and active individuals. Diagnosis is often challenging due to nonspecific symptoms, leading to delays and underestimation of prevalence. Its etiology is multifactorial, including traumatic events, iatrogenic causes, and developmental anomalies influenced by genetic factors such as *Homeobox C6* and *Paired Box 1*, which regulate scapular and acromial development. Morphological variations of the acromion, including reduced posterior coverage and altered sagittal tilt, play a critical role in the pathophysiology of posterior instability. Dynamic posterior shoulder instability can be associated with altered acromial morphology, posterior subluxation, and glenoid retroversion. In such scenarios, acromial osteotomy with posterior repositioning may be performed to enhance posterior coverage and reduce sagittal tilt. This technique allows for improved containment of the humeral head, restoration of shoulder stability, and recovery of function. Radiographic consolidation, restoration of range of motion, pain resolution, and significant improvement in functional outcome scores can be expected following successful execution of the procedure. This Technical Note emphasizes the role of acromial morphology as a critical factor in the management of posterior instability. Acromial osteotomy offers a promising strategy to optimize osseous containment and glenohumeral stability, though additional cases and long‐term follow‐up are needed to confirm reproducibility and clinical efficacy.

## Graphical abstract

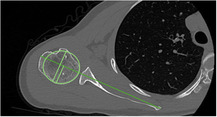

**VIDEO 1:** The patient was positioned in the beach chair. Posterior dislocation was confirmed at 30 degrees of forward elevation. A skin incision was made from the scapular spine to the anterolateral acromion. The acromial portion of the coracoacromial ligament was identified and resected. With a sterile ruler, a safe point for osteotomy was marked 2 cm above the glenoid rim. Two Kirschner wires were inserted for provisional fixation. An oblique osteotomy was then performed using an oscillating saw. The acromion was rotated 10 degrees and temporarily fixed with two Kirschner wires. Stability was tested in forward elevation, adduction with internal rotation, external rotation in aduction and abduction. After confirming proper alignment, definitive fixation was achieved with a distal radius locking plate, and final radiographs confirmed satisfactory positioning. Video content can be viewed at https://doi.org/10.1002/atn2.70026.

Posterior shoulder instability is less prevalent than its anterior counterpart. Nevertheless, recent epidemiological data indicate a rising incidence, reaching up to 24% among young and physically active individuals, significantly higher than the historically reported prevalence of approximately 5%.^1^, ^2^, ^3^, ^4^

One of the main contributing factors to this underestimation is the challenge in establishing an accurate diagnosis. While anterior instability presents with well‐defined clinical features, posterior instability often manifests as nonspecific pain and subtle symptoms, frequently leading to diagnostic delays or misclassification.^1^, ^4^

The etiology of posterior shoulder instability is multifactorial and includes traumatic events, which may cause bone loss or structural alterations of the humeral head, and iatrogenic factors,^4^ such as excessive anterior capsular tightening following anterior capsulorrhaphy,^5^ which may predispose to posterior glenoid erosion and instability. Recent studies have identified static posterior subluxation as a key contributor to the development of posterior glenoid erosion. This biomechanical alteration plays a pivotal role in the pathogenesis and progression of posterior shoulder instability.^6^

Recent studies have identified several genetic factors involved in the distinct stages of scapular development. Among these, the *Homeobox C6* gene encodes a transcription factor that contributes to the anteroposterior patterning by conferring positional identity to developing cells.^7^, ^8^, ^9^ This regulatory function is essential for the proper morphogenesis of the glenoid and coracoid processes.

In rare instances of glenoid dysplasia or hypoplasia, typically bilateral, there may be associated anomalies in both the scapula and humeral head, suggesting a developmental basis linked to genetic regulation.^10^ Notably, *Homeobox C6* has also been implicated in other conditions, including familial forms of non‐Hodgkin lymphoma, highlighting its pleiotropic roles. In addition, the *Paired Box 1* gene has emerged as a critical regulator of acromial development, supporting its role in the broader context of scapular ossification and morphogenesis.^11^

Recent studies have shown consistent and significant morphological differences in the acromion between normal shoulders and those with static posterior subluxation, as well as between clinically stable shoulders and those with dynamic posterior instability.^10^, ^11^ In particular, findings include a higher and more horizontally oriented acromion with decreased posterior coverage, which appears to diminish its stabilizing “buttress” effect. This suggests that acromial anatomy plays a critical role in the pathophysiology and biomechanical environment of posterior shoulder instability, potentially influencing glenohumeral kinematics and facilitating posterior humeral head translation.^12^

Anatomical analyses of shoulders with posterior instability and static subluxation have revealed significant deviations from normal morphology in several key parameters, including glenoid version, glenoid inclination, posterolateral acromial morphology, acromial position, and orientation. In cases of posterior instability, the acromion tends to be positioned more superiorly and assumes a more horizontal orientation, resulting in reduced posterior acromial coverage.^12^, ^13^ This reduced posterior coverage compromises the mechanical resistance to posterior humeral head translation, particularly during posterior loading or displacement, thereby contributing to both the pathogenesis and persistence of posterior instability.^13^

Notably, posterior acromial coverage appears to have a greater biomechanical impact on resisting posterior humeral head translation than glenoid retroversion alone. As a result, isolated surgical correction of glenoid version may be insufficient to fully address posterior shoulder instability.^14^, ^15^ Emerging evidence supports a more comprehensive surgical strategy that considers not only glenoid retroversion but also glenoid inclination and posterior acromial morphology as essential elements in restoring posterior glenohumeral stability and reducing the risk of recurrent instability. Importantly, glenoid osteotomy for version correction does not consistently correct static posterior subluxation, or prevent the progression of osteoarthritis, highlighting the limitations of isolated interventions.^16^, ^17^, ^18^, ^19^, ^20^

Posterior displacement of the humeral head may occur in the setting of recurrent posterior shoulder instability, even in the absence of trauma or seizure‐related events. Patients with frequent episodes often develop pain, functional limitation, and dependence on external support (sling) to prevent recurrent dislocations.

On clinical examination, posterior instability can manifest as restriction of active and passive shoulder motion, with dislocation occurring during forward elevation in the frontal plane. Reduction of the humeral head is frequently required to restore safe elevation. Range of motion may be variably affected, whereas external rotation is often preserved. The Beighton score should be assessed to exclude generalized ligamentous laxity.^21^

Magnetic resonance imaging and magnetic resonance arthrography are essential to exclude associated lesions, such as rotator cuff tears, labral injuries, or significant glenoid or humeral head bone loss (including reverse Hill‐Sachs lesions). Computed tomography is recommended to evaluate glenoid morphology and version. Friedman's method^22^ can be used to calculate glenoid retroversion by measuring the angle between the glenoid line (connecting anterior and posterior rims) and the scapular axis. Hoenecke's method^23^ may also be applied to reduce variability related to scapular anatomy, particularly in developmental dysplasia or posterior glenoid erosion.

Posterior humeral head subluxation can be quantified using the glenohumeral index and the scapulohumeral index, both of which provide objective and reproducible measures of posterior displacement. These indices support surgical decision‐making by defining the degree of static or dynamic posterior subluxation.^24^

Radiographic analysis should also include posterior acromial coverage, the critical shoulder angle (CSA), and sagittal acromial tilt. The CSA is measured on a true anteroposterior radiograph of the shoulder. A line is drawn from the superior to the inferior bony margins of the glenoid. A second line is then drawn from the inferior glenoid margin to the most lateral point of the acromion. The angle formed between these 2 lines represents the CSA.^25^

Sagittal tilt is measured on a scapular Y‐view. A reference line is drawn along the longitudinal axis of the scapula, and a second line is drawn along the undersurface of the acromion. The angle formed between these 2 lines defines the sagittal acromial tilt. The normal value is approximately 53°. Values greater than this indicate a flatter acromion with reduced posterior coverage. Decreasing this angle improves acromial biomechanics by creating a steeper orientation, which enhances posterior containment of the humeral head.^10^

Posterior acromial coverage is assessed on a scapular Y‐view. A line is drawn from the center of the humeral head to the inferior glenoid margin, and a second line is drawn from the same point to the most posterior extent of the acromion. The angle between these 2 lines quantifies the degree of posterior coverage. The normal value for posterior acromial coverage is approximately 63°. When the measured angle is below this threshold, posterior coverage should be increased to improve humeral head containment and enhance glenohumeral stability. Greater coverage is thought to enhance posterior containment of the humeral head and contribute to glenohumeral stability.^10^, ^13^

These morphological parameters are valuable for understanding structural contributors to posterior instability. For instance, excessive sagittal tilt and reduced posterior coverage can compromise humeral head containment, while CSA variations may influence overall glenohumeral stability.^23^, ^25^ Collectively, these radiographic measurements establish the anatomical foundation for planning surgical correction, such as acromial osteotomy and posterior repositioning.

## SURGICAL TECHNIQUE

### Patient Evaluation, Imaging, and Indications

Dynamic posterior shoulder instability should be carefully evaluated through clinical examination, functional scoring, and imaging studies. Functional assessment typically includes standardized outcome measures, such as the American Shoulder and Elbow Surgeons score, the Western Ontario Shoulder Instability index, and the visual analog scale for pain. These tools provide quantitative assessment of baseline disability and allow for monitoring of postoperative improvement.

Clinical examination should assess range of motion in multiple planes, documenting forward elevation, external and internal rotation, and abduction. Particular attention should be given to posterior subluxation or dislocation occurring during forward elevation, as this clinical sign is highly relevant for surgical indication.

**FIGURE 1:**
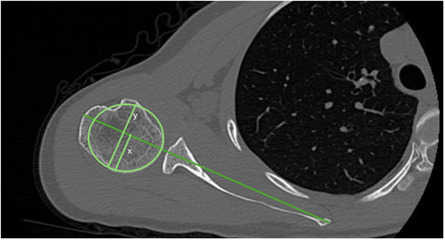
Axial computed tomography scan of the shoulder demonstrating the method for calculating the Posterior Subluxation Index (PSI) using Friedman's line. Measurement Y represents the anteroposterior diameter of the humeral head, and measurement X represents the distance from the anterior margin of the humeral head to Friedman's line. The PSI is calculated as (X/Y) × 100. In this case, the PSI was approximately 68.8%, indicating significant posterior humeral head subluxation.

Magnetic resonance imaging and magnetic resonance arthrography are recommended to rule out associated pathology such as labral injuries or rotator cuff tears, while computed tomography provides accurate quantification of glenoid version using standardized methods, including Friedman and Hoenecke techniques (Figure 1).^22^, ^23^ Posterior subluxation of the humeral head can be quantified with the glenohumeral and scapulohumeral indices, and complementary radiographic parameters, such as posterior acromial coverage, sagittal tilt, and the CSA, should be assessed to determine morphological contributors to instability.

Indications for acromial osteotomy and posterior repositioning include recurrent dynamic posterior instability with morphological alterations of the acromion, excessive sagittal tilt, or insufficient posterior coverage, as shown on imaging and clinical examination.

**FIGURE 2:**
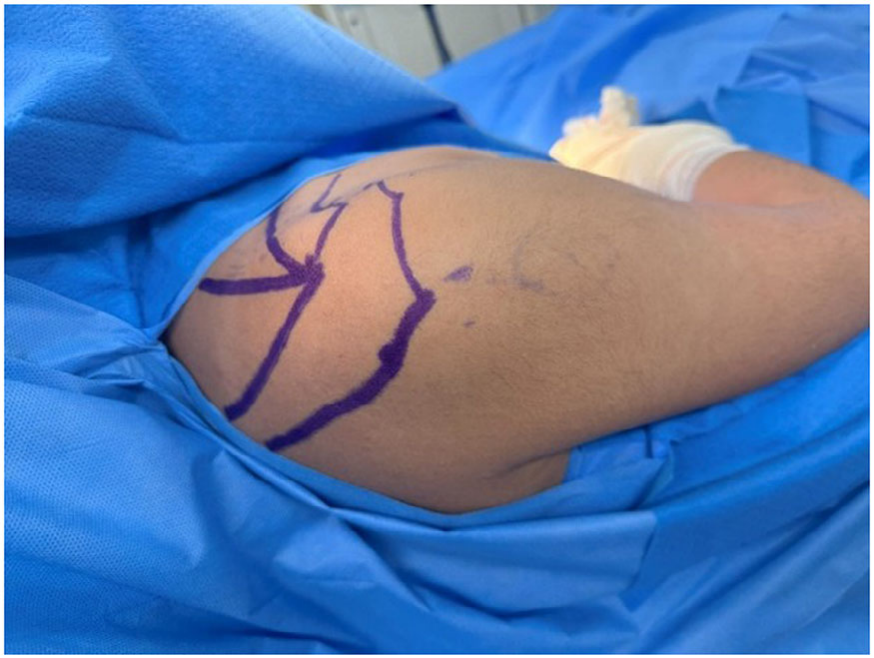
Patient is positioned in the beach‐chair position; anatomical landmarks of the shoulder. The outlines indicate the acromion and scapular spine.

The procedure is performed under general anesthesia combined with a brachial plexus block, with the patient positioned in the beach‐chair position at a 30° inclination. Preoperative skin markings were performed on the shoulder, identifying key anatomical landmarks, such as the acromion and scapular spine for surgical orientation (Figure 2).

**FIGURE 3:**
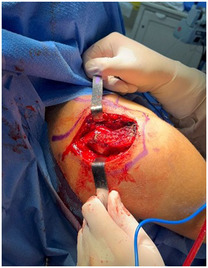
Intraoperatively, a partial detachment of the posterior musculature was noted, involving fibers of the deltoid and trapezius muscles, which were carefully mobilized to optimize exposure while preserving tissue integrity.

**FIGURE 4:**
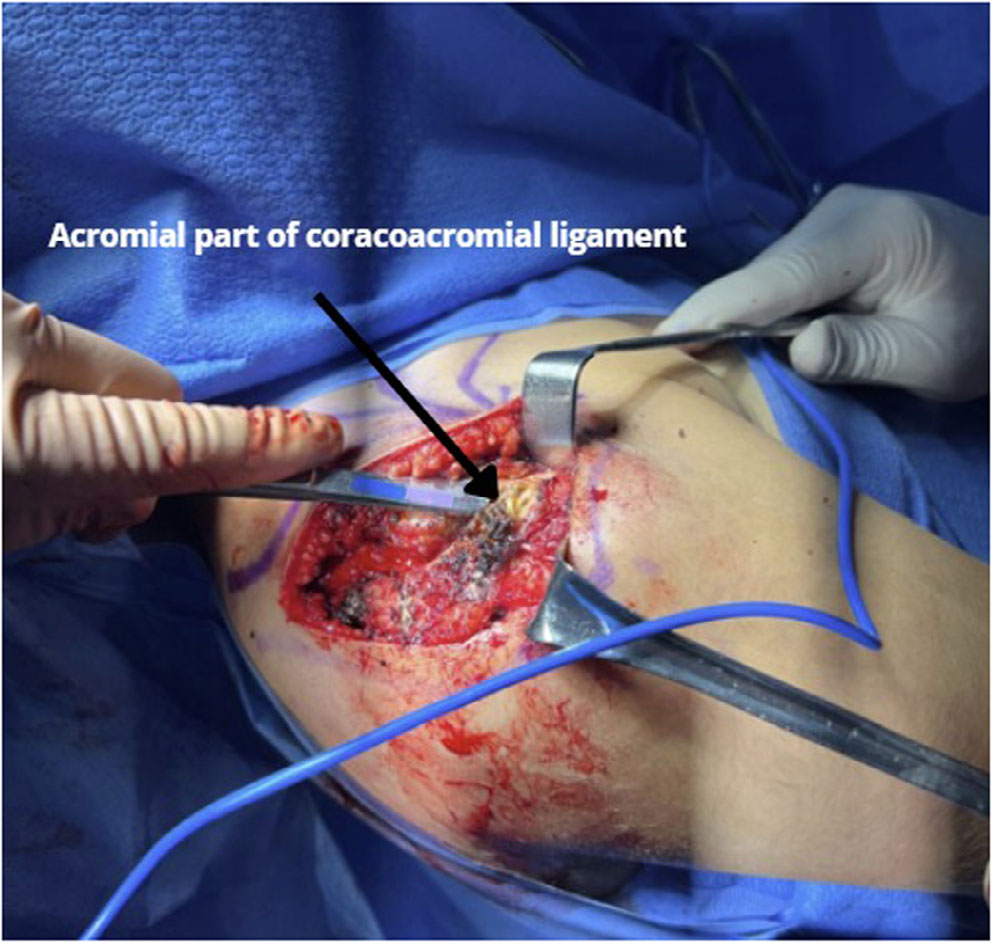
Patient in beach‐chair position; posterior view. The black arrow indicates the acromial portion of the coracoacromial ligament, which is resected to allow posterior rotation of the acromion.

A posterior surgical approach is utilized, with a skin incision made along the spine of the scapula and extended toward the posterolateral border of the acromion (Video 1). The trapezius is partially elevated from the scapular spine, while the posterior and middle deltoid fibers are carefully detached from their acromial origin, allowing optimal exposure of the posterior scapular region (Figure 3). The coracoacromial ligament is identified (Figure 4) and carefully released from its acromial attachment, a critical maneuver that facilitates posterior rotation of the acromion, thereby increasing posterior acromial coverage of the humeral head and contributing to enhanced posterior shoulder stability.

**FIGURE 5:**
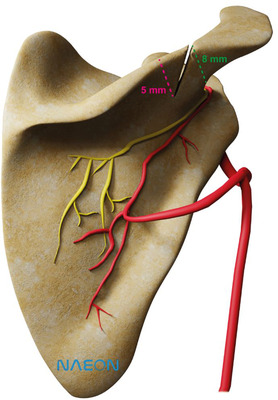
Schematic representation of the planned trapezoidal acromial osteotomy, illustrating measurements of 8 mm at the posterolateral edge and 5 mm medially. The planned cut respects a 2‐cm safe distance from the posterior glenoid rim, minimizing risk to adjacent neurovascular structures. The image also depicts the anatomical relationship between the acromion, scapular spine, and surrounding vascular and neural elements, emphasizing the importance of surgical precision.

**FIGURE 6:**
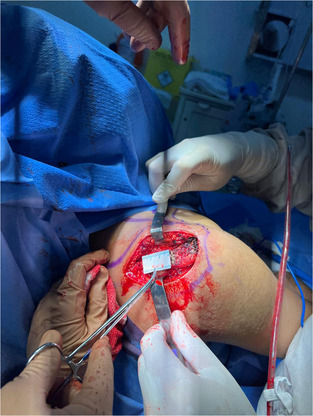
Patient in beach‐chair position; posterior view. Using a sterile ruler with 2‐cm markings, we place the lateral edge close to the posterior rim of the glenoid, thereby establishing our safety point for the osteotomy.

**FIGURE 7:**
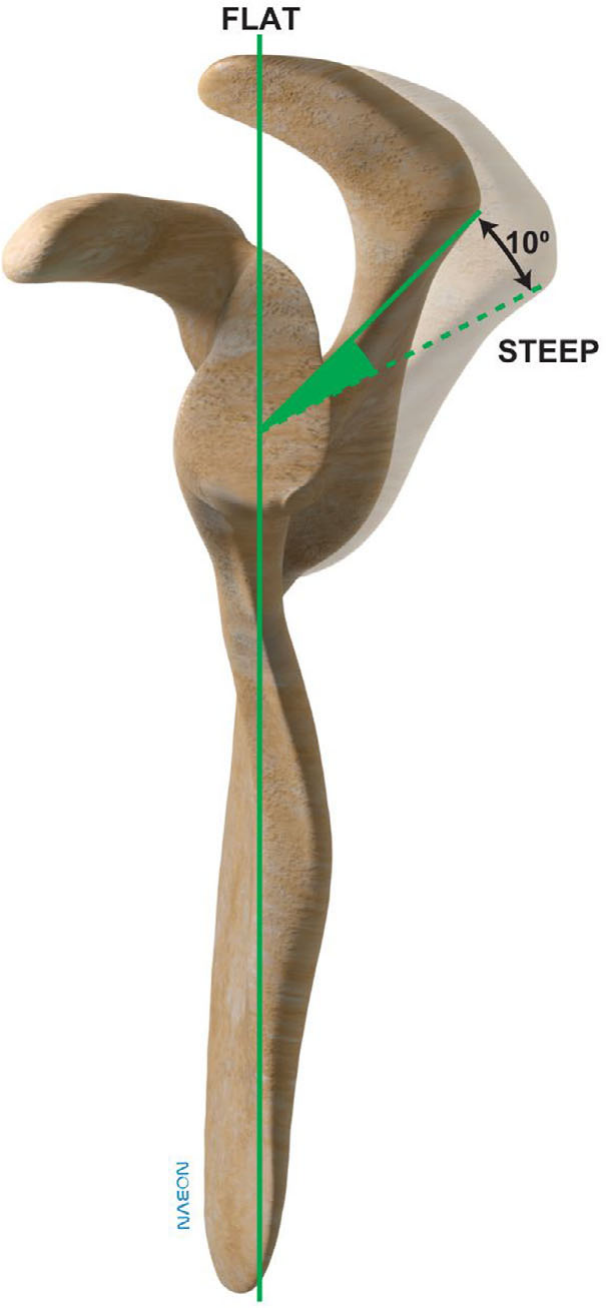
Schematic illustration of the acromial osteotomy showing the posterior rotation of 10° performed to convert a flat acromion into a steeper configuration, thereby improving posterior humeral head coverage.

Following soft tissue mobilization, osteotomy planning is carried out. A trapezoidal acromial osteotomy is outlined, measuring 8 mm in height at the posterolateral edge and 5 mm medially, enabling controlled lateral and posterior translation of the acromion (Figure 5). The entry point for the cutting guide is precisely marked, maintaining a 2‐cm distance from the posterior glenoid rim to the acromion, thereby ensuring procedural safety and minimizing the risk of glenoid injury (Figure 6). This precaution is particularly relevant as the suprascapular nerve and artery traverse posteriorly at a mean distance of 1.8 cm from the posterior glenoid border, reducing the risk of iatrogenic injury. The procedure aims to correct sagittal tilt while increasing posterior acromial coverage, thereby optimizing humeral head containment, and the acromion is rotated posteriorly by approximately 10°, thereby enhancing posterior coverage and decreasing sagittal tilt (Figure 7).

**FIGURE 8:**
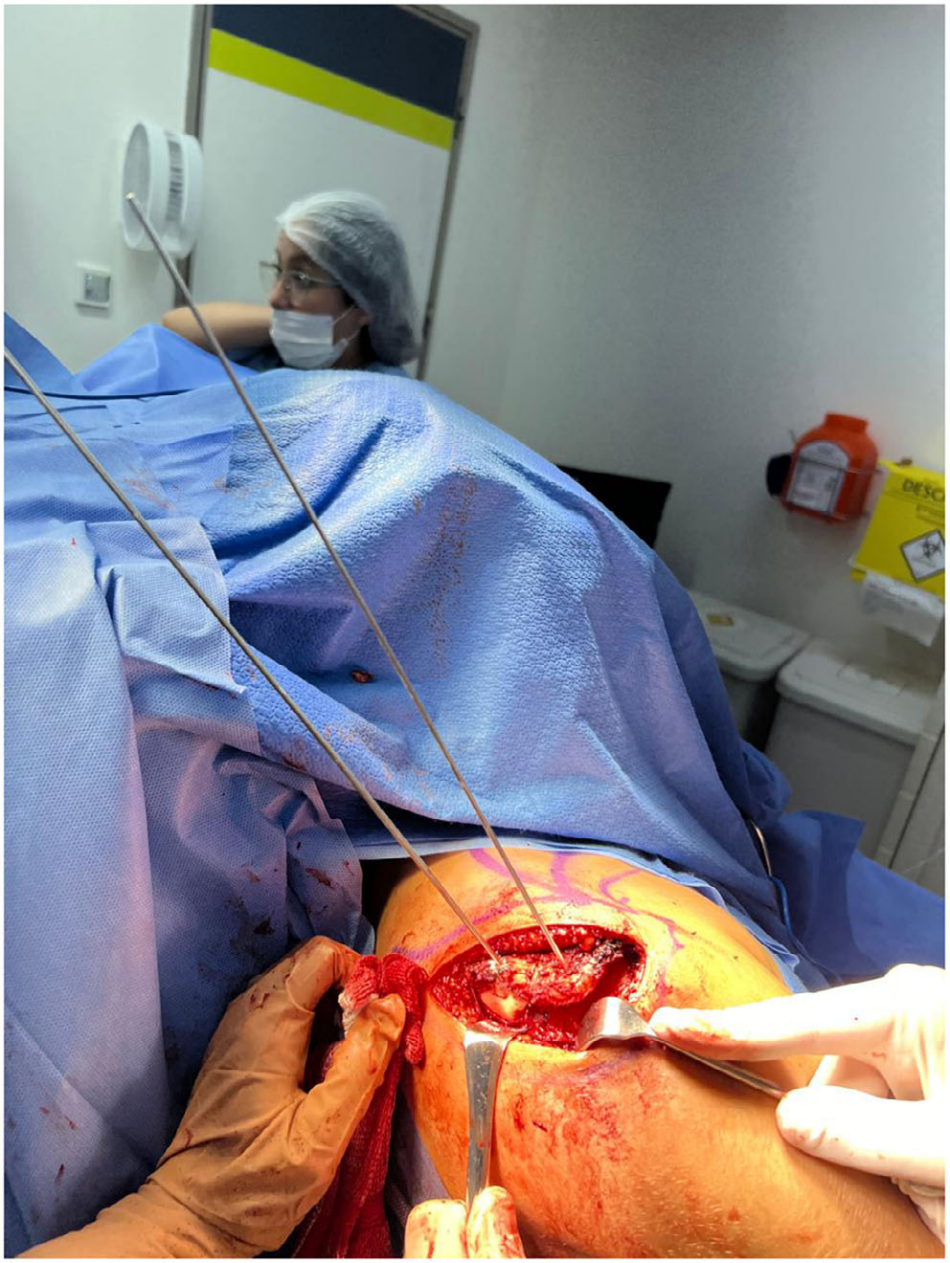
Patient in beach‐chair position; posterior view. Fixation with two 2.0‐mm Kirschner wires is performed to ensure safe execution of the acromial osteotomy, delineating the secure region for the osteotomy.

**FIGURE 9:**
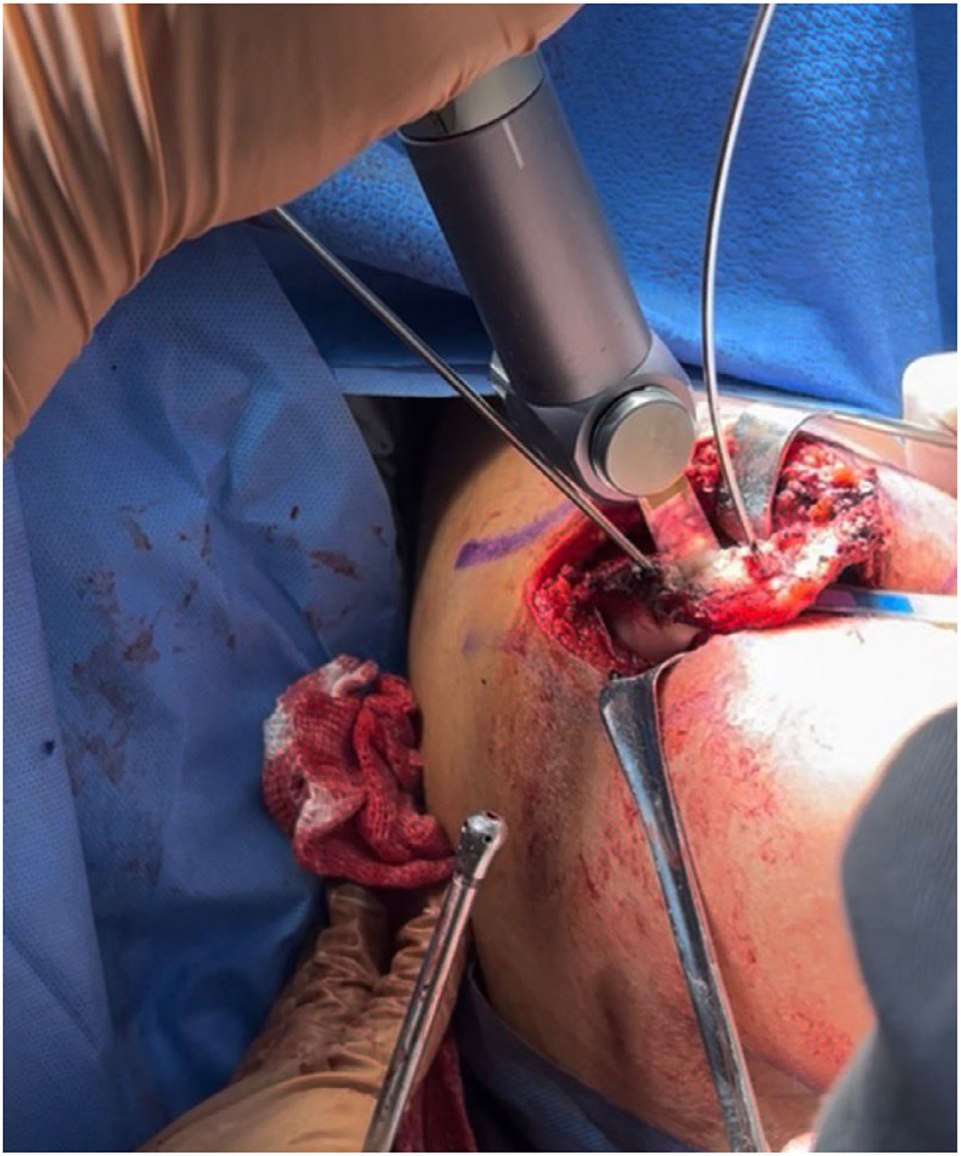
Patient in beach‐chair position; posterior view. The osteotomy is performed from superior to inferior, an oblique fashion using an oscillating saw.

**FIGURE 10:**
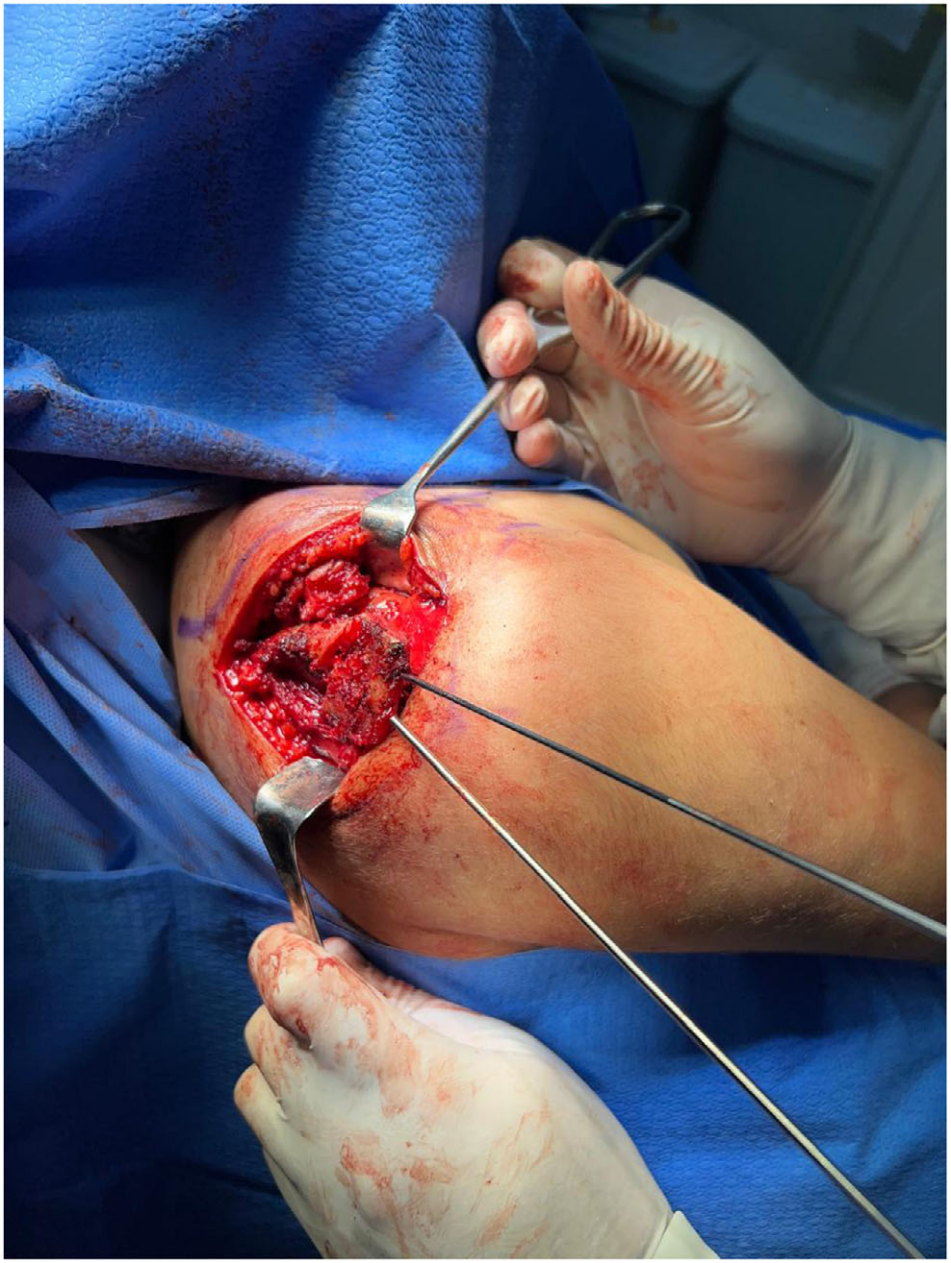
Patient in beach‐chair position; posterior view. After the osteotomy, provisional fixation is performed with two 2.0‐mm Kirschner wires solely to test posterior stability and to verify whether 10° of posterior rotation are sufficient to improve posterior coverage.

With the safe osteotomy position identified, two 2‐mm Kirschner wires are inserted (Figure 8) medially and laterally for provisional stabilization, securing the acromion in its native position prior to the osteotomy. The osteotomy is performed in an oblique fashion using an oscillating saw between the 2 Kirschner wires (Figure 9). A controlled lateral and posterior translation of the acromion is then performed, resulting in increased posterior coverage and a reduction in sagittal tilt. The acromion is temporarily fixed with 2 additional Kirschner wires to confirm optimal positioning prior to definitive fixation (Figure 10).

**FIGURE 11:**
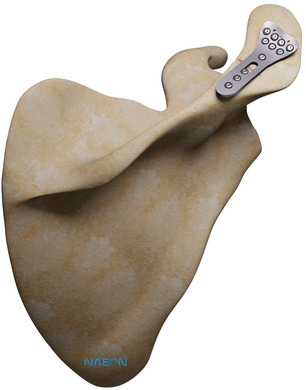
Posterior schematic view showing plate positioning after acromial osteotomy with medial eccentric screw compression and secure fixation using 3 cortical screws and distal locking screws.

**FIGURE 12:**
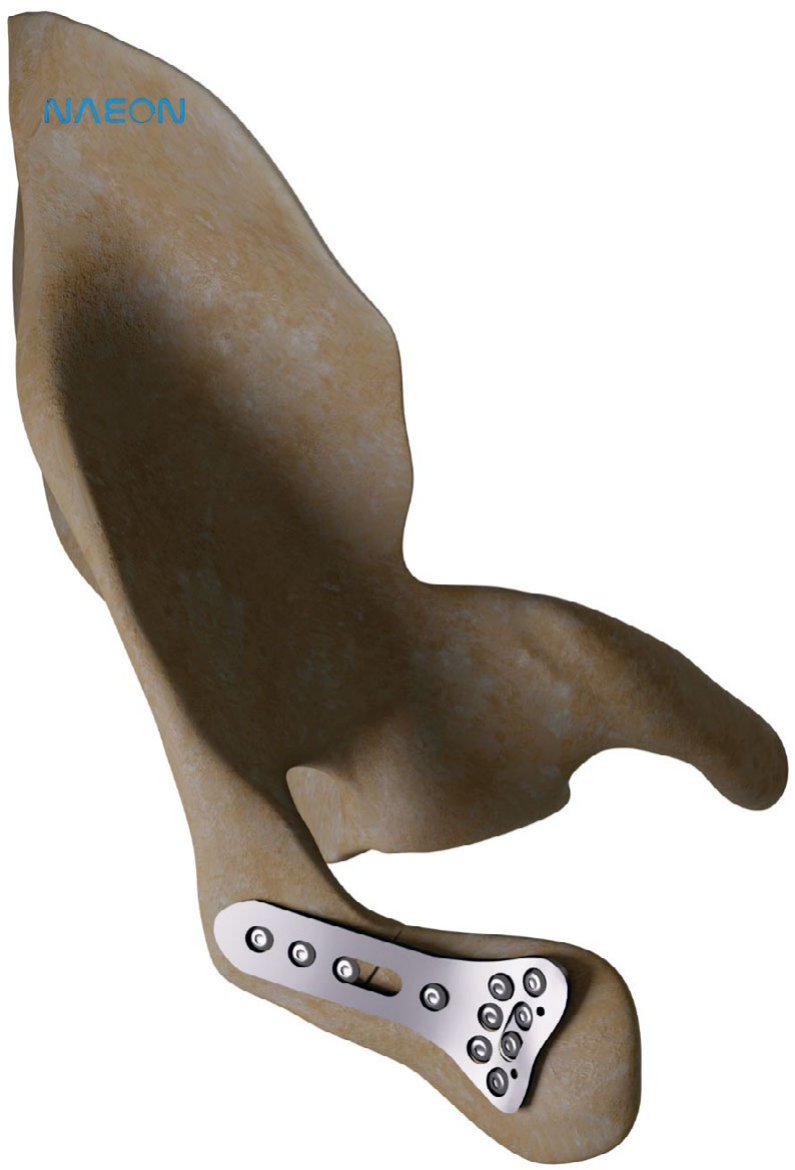
Schematic superior view of the shoulder demonstrating proper plate positioning after acromial osteotomy. Compression at the osteotomy site is achieved with a medial eccentric screw, providing stable fixation. The construct includes 3 cortical screws proximally and distal locking screws for additional stability.

**FIGURE 13:**
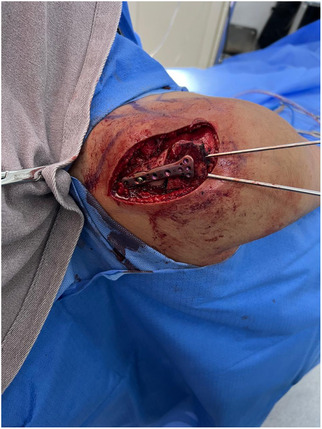
Patient in the beach‐chair position with posterior view of the shoulder. The plate is positioned on the acromion, with initial screw fixation being performed. Provisional Kirschner wires are in place to maintain the reduction and prevent displacement during plate fixation.

The plate is positioned on the superior aspect of the acromion. Fixation begins with placement of the eccentric screw medially to achieve compression at the osteotomy site. Subsequently, 2 additional cortical screws are inserted, and the provisional Kirschner wire is removed. Distal locking screws are then placed to complete fixation. Particular care must be taken regarding screw length to avoid cortical perforation and potential rotator cuff damage during shoulder elevation and abduction (Figures 11, 12, 13).

Final stabilization of the osteotomy is achieved using a distal radius locking plate, providing rigid fixation and maintaining the desired correction.

After plate placement, reinsertion of the posterior deltoid and trapezius muscles is meticulously performed using transosseous FiberWire sutures, restoring soft tissue continuity and muscular integrity. A Hemovac drain is placed in the posterior surgical field to minimize hematoma formation and is removed 24 hours postoperatively. Pearls and pitfalls of the described procedure are presented in Table 1. The patient is immobilized in a regular sling for a period of 8 weeks postoperatively to protect the osteotomy site and allow for adequate soft tissue healing.

**TABLE 1:** Pearls and Pitfalls of Acromial Osteotomy

**Pitfalls**
Resection of the acromial portion of the coracoacromial ligament is required to allow acromial rotation
Temporary fixation with Kirschner wires should be used to ensure proper alignment
Placement of a suction drain is recommended to prevent hematoma formation
The distal screws of the plate should not be excessively long to avoid protrusion, which may cause impingement on the rotator cuff

**FIGURE 14:**
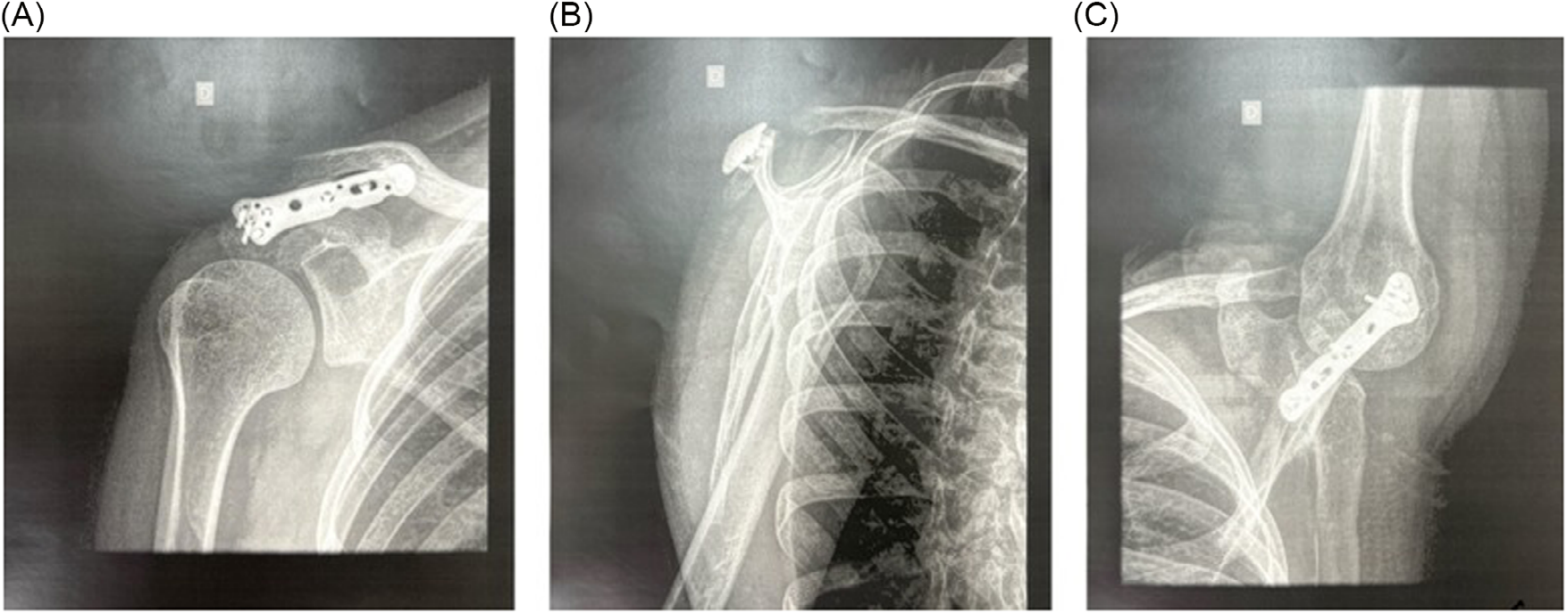
Postoperative radiographic images demonstrating acromial fixation using a distal radius locking plate. (A) Anteroposterior view showing the position of the plate along the posterior acromion. (B) Scapular Y view confirming proper posterior translation and alignment, without causing acromioclavicular joint issues or displacement. (C) Axillary lateral view demonstrating stable fixation and increased posterior acromial coverage over the humeral head.

**FIGURE 15:**
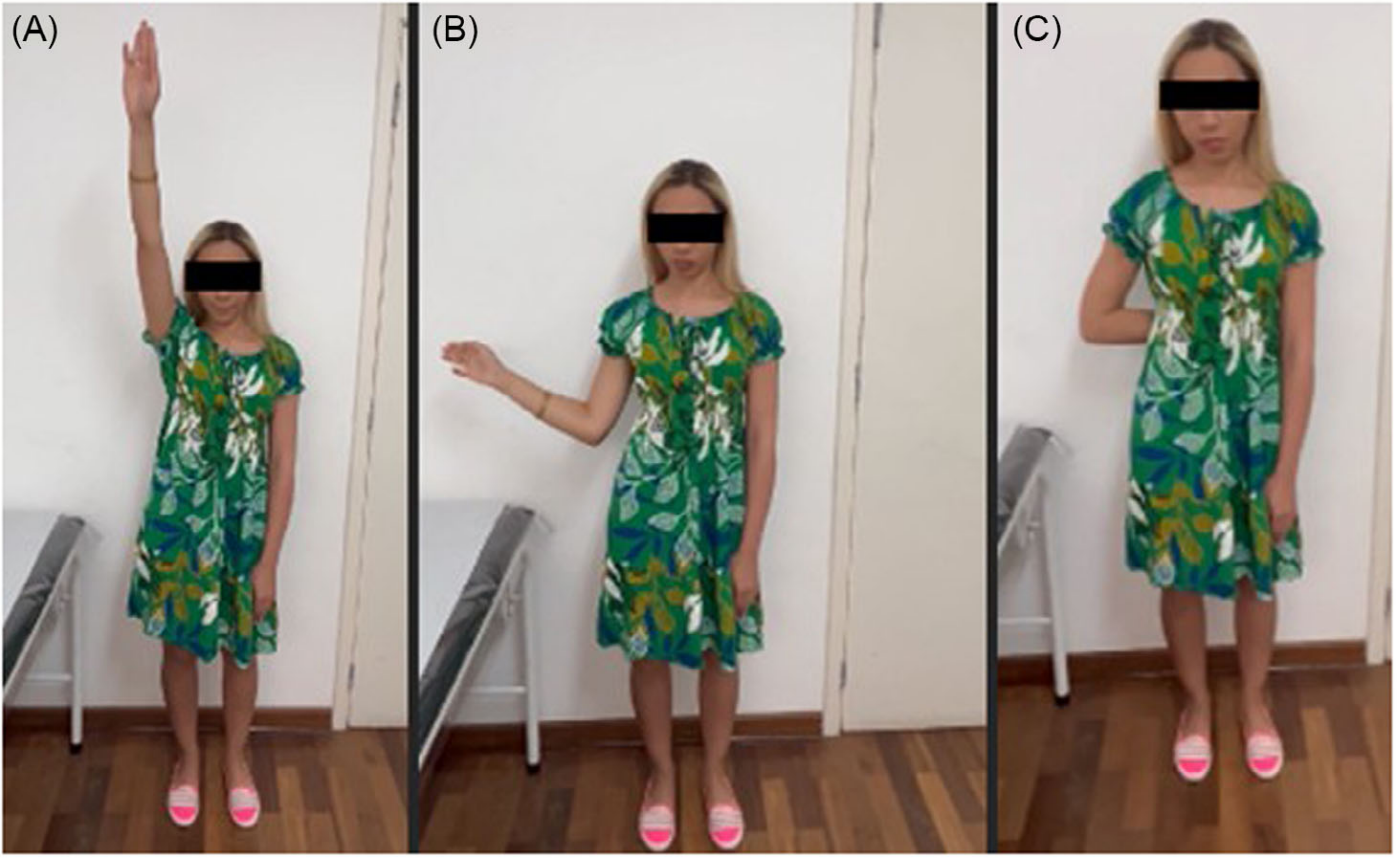
Postoperative clinical evaluation of shoulder function after 12 months follow‐up. Sequential images show the following: (A) Active forward elevation is completed, (B) external rotation with the arm in adduction, and (C) internal rotation with the hand positioned in the back. *Note*: Preserving full range of motion and preventing further episodes of posterior dislocation. *All images were obtained with the full consent and authorization of the patient, in accordance with institutional ethical guidelines and patient privacy regulations.

At the 1‐year follow‐up, radiographic evaluation confirmed complete bone consolidation, and both passive and active range of motion were fully restored. The patient showed no movement restrictions and experienced no loss of muscular strength, indicating successful functional recovery (Figure 14 and 15).

## DISCUSSION

Posterior shoulder instability is increasingly recognized as a significant cause of shoulder pain and dysfunction.^1^, ^24^, ^26^ Several anatomical and biomechanical factors, most notably posterior capsulolabral tears, glenoid retroversion, and posterior glenoid bone loss, have been consistently associated with reduced resistance of the humeral head to posteriorly directed forces. Although initial management often involves conservative measures, surgical intervention is indicated when nonoperative treatment fails.^15^, ^16^, ^17^, ^19^, ^20^

Multiple surgical strategies have been described, including arthroscopic or open capsulolabral repair,^26^, ^27^ glenoid osteotomies (such as the J‐graft technique),^19^, ^20^ and posterior glenoid bone augmentation procedures,^15^, ^16^, ^24^ either in isolation or in combination. Despite these advances, clinical outcomes remain challenging. Reported failure rates reach up to 35% for isolated capsulolabral repairs,^26^, ^27^ as high as 73% for open posterior bone block procedures,^15^, ^16^, ^24^ and up to 33% for glenoid osteotomies at long‐term follow‐up.^23^, ^24^, ^26^

Recently, growing attention has been directed toward the role of acromial morphology in posterior instability and posterior glenoid bone loss. This emerging evidence highlights the importance of considering scapular and acromial anatomy as contributing factors in the pathophysiology of posterior shoulder instability,^28^, ^29^, ^30^, ^31^, ^32^, ^33^ and may open new perspectives for surgical strategies beyond traditional glenoid‐ or capsulolabral‐focused approaches.

This Technical Note describes the application of acromial osteotomy in the surgical treatment of dynamic posterior shoulder instability. Advantages and disadvantages of the procedure are presented in Table 2. The primary objective of this technique is to restore the patient's full range of motion, particularly forward elevation, while preventing posterior dislocation during movement. Additionally, it aims to restore posterior acromial coverage and optimize sagittal acromial tilt, thereby enhancing the biomechanical resistance to posterior translation of the humeral head. A deficient posterior acromial morphology, characterized by short lateral extension of the acromial roof, reduced external rotation, flattened sagittal inclination, and limited posterior glenoid coverage, creates an inherently unstable anatomical configuration.^10^, ^13^ In this context, there is increased reliance on dynamic stabilizers, particularly the rotator cuff and scapular muscles, to maintain humeral head centering within the glenoid. The excessive functional demand placed on these muscles predisposes patients to fatigue‐related instability episodes, as the osseous constraints are insufficient to provide adequate posterior stabilization.^10^, ^31^, ^33^

**TABLE 2:** Advantages and Disadvantages of Acromial Osteotomy

**Advantages**	**Disadvantages**
Prevents progression of glenohumeral osteoarthritis	Possible need for plate removal due to superficial location of the acromion
Corrects shoulder biomechanics, protecting the rotator cuff	
Does not preclude future shoulder arthroplasty procedures	
Low cost compared with alternative procedures	

Conversely, an acromion with greater posterior inclination and increased posterior glenoid coverage may enhance static containment of the humeral head, reducing the necessity for compensatory muscular effort. In contrast, a flatter acromial configuration with diminished glenoid support offers less resistance to posterior translation, further compromising joint stability.^10^, ^31^, ^32^ These morphological variations directly alter the mechanical vectors and functional dynamics of the deltoid and rotator cuff, thereby influencing both force distribution and dynamic joint mechanics.^31^, ^32^

By modifying acromial orientation and enhancing posterior coverage, acromial osteotomy offers a biomechanical approach to address the bony deficiencies that contribute to posterior shoulder instability.^33^ This technique has the potential to reduce reliance on soft tissue reconstructions alone, providing a structurally integrative and potentially more durable solution for selected cases of dynamic posterior instability, particularly in patients with inadequate posterior osseous containment.^10^, ^31^, ^32^, ^33^

Recent studies have emphasized the critical role of the acromion in controlling posterior humeral head translation. In both normal shoulders and those classified as Walch B1, the acromion maintains full contact with the humeral head at 60° of elevation, suggesting a stabilizing biomechanical interaction during overhead motion.^6^, ^28^

This relationship may help explain the limited success of isolated glenoid osteotomy or posterior J‐bone grafting in fully preventing posterior humeral head translation. While these procedures effectively address glenoid retroversion and posterior bone loss, they do not modify acromial morphology, which has been shown to play a substantial role in posterior stabilization.^13^

Given the acromion's involvement in humeral head containment, anatomical variations in posterior acromial coverage may contribute to persistent instability, even after successful glenoid correction. These findings underscore the importance of a comprehensive surgical approach that includes acromial realignment in select cases, aiming to optimize the osseous architecture for enhanced posterior shoulder stability.^13^, ^28^

By modifying acromial morphology, the described technique aims to optimize posterior acromial coverage and sagittal tilt, thereby improving humeral head containment and reducing reliance on dynamic stabilizers. Despite these encouraging preliminary findings, additional clinical cases and long‐term follow‐up studies are necessary to confirm the technique's efficacy, assess its reproducibility, and determine its impact on long‐term joint preservation.

## DISCLOSURES

The authors (R.M., J.C.G., M.B., A.C., L.F., M.R., M.M.) declare that they have no known competing financial interests or personal relationships that could have appeared to influence the work reported in this paper.
